# The complete mitochondrial genome of *Anneissia intermedia* (Crinoidea: Comatulida: Comatulidae)

**DOI:** 10.1080/23802359.2021.1933633

**Published:** 2021-05-27

**Authors:** Philjae Kim, Taekjun Lee, Sook Shin

**Affiliations:** a Division of Ecological Conservation, Bureau of Ecological Research, National Institute of Ecology, Korea; b Department of Animal Biotechnology and Resource, Sahmyook University, Seoul, Korea

**Keywords:** Echinodermata, crinoid, phylogeny, mitogenome, *Anneissia intermedia*

## Abstract

*Anneissia intermedia* (A.H. Clark, 1916) is a common crinoid found in waters along the coastlines of China, Japan, and Korea. In this study, we determined the complete mitogenome of *A. intermedia*. The genome was found to be 15,874 bp in length and consists of 13 protein-coding genes, 22 tRNAs, and 2 rRNAs. With the exception of *Antedon mediterranea*, the gene order and genetic characteristics of the *A. intermedia* mitogenome are identical with those of the mitogenomes of other crinoids. The complete mitogenome of *A. intermedia* will contribute to enhance our understanding of the phylogeny of crinoids.

The genus *Anneissia* consists of nine species distributed over a wide range of the Indo-western Pacific Ocean, including Australia, New Zealand, New Caledonia, China, Japan, and Korea (Clark and Rowe 1971; Shin 2013; Messing 2020). Although the different species of *Anneissia* are among the most abundant crinoids in the Pacific region, to date, the sequencing of a complete mitogenome has been reported for only a single species, namely, *A. pinguis*. In this study, we augment this sole representative of the genus *Anneissia* by determining the complete mitochondrial genome of *A. intermedia*. Specimens of *A. intermedia,* which were identified based on morphological observations, were collected by SCUBA diving at a depth of 14 m in waters off the coast of Busan, Korea, on 25 September 2020 (35.037028 N, 129.091806 E). Voucher specimens and mitochondrial DNA (mtDNA) samples have been deposited at the Marine Echinoderm Resources Bank of Korea (http://www.syu.ac.kr; Taekjun Lee, leetj@syu.ac.kr) under the voucher number MERBK-C-00307. mtDNA extraction, amplification, and sequencing were performed in accordance with the methods described by Lee and Shin (2018). The complete mitogenome was annotated using Geneious ver. 11.1.5 (Biomatters Ltd, Auckland, New Zealand), and transfer RNAs (tRNAs) were identified and gene locations determined as described by Lee and Shin (2019). Phylogenetic analysis was performed using 24 complete echinoderm mitogenomes [nine crinoids (including *A. intermedia*), four asteroids, four ophiuroids, four echinoids, and three holothuroids], with those of two hemichordates (*Balanoglossus carnosus* and *B. clavigerus*) being used as an outgroup. Mitogenome sequences were aligned using MAFFT (Katoh and Standley 2013) and the mitogenome dataset was analyzed based on the maximum likelihood (ML) method using RAxML 8.2 (Stamatakis 2014). The best-fit substitution was estimated using jModelTest 2.1.1 (Guindon and Gascuel 2003; Darriba et al. 2012) for the nucleotide dataset of 13 protein-coding genes (PCGs). For ML analyses, bootstrap resampling was performed using the rapid option with 1000 iterations.

The complete mitogenome of *A. intermedia* (GenBank accession No. MW376476) is 15,874 bp in length and contains 13 PCGs, 22 tRNA genes, and two rRNA genes. With the exception of that of *Antedon mediterranea*, the gene order and direction of PCGs and rRNAs were found to be identical to those of the complete mitogenomes of other comatulid species. All PCGs contain an ATG initiation codon (methionine), and the termination codons are generally either TAA or TAG, with that of cytochrome *b*, which terminates with (GAG)+T (glutamic acid), being the only exception.

In the constructed phylogenetic tree, the nine complete mitogenomes of crinoids clearly established a monophyletic clade (Figure 1), within which, *A. intermedia* clusters with *A. pinguis* and *Phanogenia gracilis*, belonging to the same family, Comatulidae (Figure 1). The sequencing of the complete mitogenome of *A. intermedia* will provide a basis for further phylogenetic studies on crinoids.

**Figure 1.**
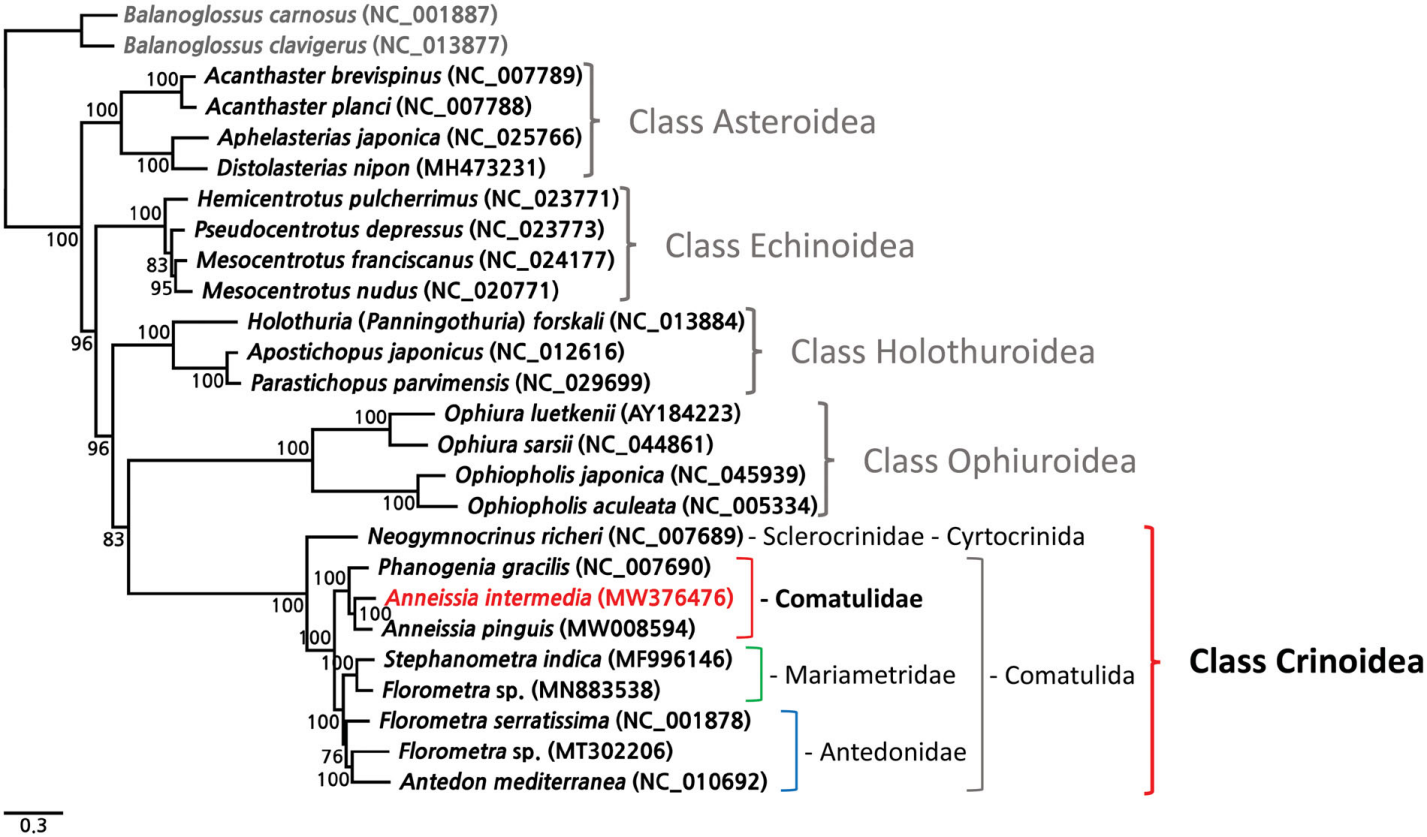
A phylogenetic tree constructed using the maximum likelihood (ML) method based on the nucleotide sequences of 13 protein-coding genes of nine crinoids, including *A. intermedia* (MW376476), other echinoderms, and two outgroup species (*B. carnosus* and *B. clavigerus*). Bootstrap support values were indicated on each node.

## Data Availability

The data that support the findings of this study are available at https://www.ncbi.nlm.nih.gov/, accession number MW376476. The associated BioProject, Bio-Sample, and SRA numbers are PRJNA715061, SAMN18325186, and SRR13996249, respectively.
